# A Case of Pulmonary Cement Embolism Managed through Symptomatic Treatment

**DOI:** 10.1155/2020/2425973

**Published:** 2020-06-04

**Authors:** Alex R. Waler, Kyle J. Sanchez, Amay A. Parikh, Okorie N. Okorie

**Affiliations:** ^1^University of Central Florida College of Medicine, Orlando, FL, USA; ^2^Advent Health, Orlando, FL, USA

## Abstract

**Objective:**

This case describes symptomatic pulmonary cement embolism as a rare postvertebroplasty complication and highlights its critical yet ill-defined management.

**Background:**

Pulmonary cement embolism (PCE) is a feared complication of vertebroplasty in the treatment of vertebral fractures. While the majority of PCEs are asymptomatic, symptomatic PCEs often present with chest pain, tachycardia, signs of severe respiratory distress, and death. Computer tomography angiogram (CTA) allows visualization of cement within the pulmonary vasculature. Despite the well-established risk of PCE, clinical management is unclear with limited research on treatment options. Reported treatments include anticoagulation, embolectomy, CPR, and supportive care and observation. *Report*. We report the case of a 75-year-old woman who experienced shortness of breath, tachypnea, tachycardia, hypertension, and hypoxemia five days following a corrective surgery for a compression fracture of L3 with pedicle screw fixation, fusion of L2 through L4, and L2 vertebral body cement augmentation with polymethyl methacrylate.

**Results:**

Breath sounds were diminished bilaterally with respiratory alkalosis and hypoxemia evident on arterial blood gas. CTA revealed intravasated cement throughout the right lung, including the pulmonary artery and upper and middle lobar arteries. The proposed mechanism is embolization of cement particles from the lumbar veins, which also showed intravasation. Due to the inorganic nature of the occluding material, the use of a thrombolytic agent was ruled against. Treatment included bronchodilators, 3 L of oxygen via nasal cannula, and prophylactic antibiotics, pulmonary toilet, and incentive spirometry. Symptomatic management was continued until she was discharged from the hospital in a stable condition.

**Conclusions:**

Postvertebroplasty pulmonary cement embolisms can be managed conservatively, without the use of anticoagulant or thrombolytic agents. This case illustrates a variation of care for this rare presentation and adds to the sparse literature on the management of PCEs.

## 1. Introduction

Vertebroplasty is commonly used to treat vertebral fractures. During this procedure, polymethyl methacrylate (PMMA) cement is injected under fluoroscopic or computerized tomography (CT) guidance resulting in the reduction or termination of pain in 70% to 90% of cases [1]. During the procedure, the cement maintains viscosity for an average of 20 minutes, according to the commercial product reports on PMMA [2]. The pliability of the cement during this time is responsible for its occasional extravasation into the venous system and spinal canal, which is a known complication of vertebroplasties. While leakage of the cement outside the vertebral body occurs in up to 65% of cases [3], pulmonary cement embolism (PCE) has been recorded to occur much less often, varying from 4.6% to 23% of cases [4, 5]. PCEs are believed to originate from cement extravasation into the basivertebral veins, which drain into the inferior vena cava (IVC) and ultimately lodge in the pulmonary capillaries. Cement infiltration of the IVC is currently the only significant risk factor associated with PCE [5]. However, the vascular anatomy, the fracture pattern, the force with which the cement is applied, and the viscosity of the cement likely play a crucial role in the development of PCE [3, 6].

While the vast majority of PCEs are asymptomatic [7], clinical presentation can vary, with dyspnea being the most common complaint in symptomatic patients. Other reported symptoms include tachypnea, tachycardia, cyanosis, chest pain, cough, hemoptysis, sweating, and even death postvertebroplasty [6]. These symptoms often arise within 48 hours of vertebroplasty, but delayed onset of dyspnea has also been reported multiple days after the surgery or after discharge [3]. Diagnosis of PCE can be made solely on chest X-ray with high density markings in the pulmonary artery distribution [4, 8]. However, the majority of clinicians opt for a CT scan for its improved detail. The cement emboli appear on CT scans as radiodensities of more than 1,000 HU (Hounsfield units), which differentiates this condition from the hypodensities created by thrombotic emboli [8]. With the majority of PCE being asymptomatic, some clinicians recommend ordering a routine chest X-ray and/or CT scan on all patients following vertebroplasty [7, 8].

While the diagnosis of symptomatic PCE can be easily confirmed by imaging, there are no standard guidelines on the management of PCE, and the treatment selection is often dependent on the severity of each individual case [7]. Treatment options include anticoagulation, embolectomy, CPR, and supportive care and observation. Anticoagulation is the elected treatment reported in most symptomatic cases [9], while asymptomatic cases are often treated conservatively with symptomatic management and close clinical observation [6]. For most symptomatic CFE cases, clinicians follow the standard treatment guidelines for thromboembolic pulmonary emboli by initiating anticoagulation with intravenous or subcutaneous heparin to bridge long-term oral warfarin use for three to six months [6]. However, some case reports have shown that embolectomy drastically reduces patient morbidity and mortality [9, 10]. Postembolectomy, Tozzi et al. reported that the resected cement was almost entirely coated by thrombus despite the coadministration of anticoagulation [9], which may argue the futility of anticoagulation. Still, others contend that the risk of death postembolectomy for CFE may not be worth its potential benefit [11]. Due to the mechanically occlusive and inorganic nature of cement emboli, the use of anticoagulation is based on physician preference [12].

## 2. Case Presentation

This patient was a 75-year-old Caucasian female with no known comorbidities who experienced shortness of breath, tachypnea, tachycardia, hypertension, and hypoxemia five days after an elective, corrective surgery for a compression insufficiency fracture of L3 with concurrent revision of previous vertebral hardware.

**Figure 1:**
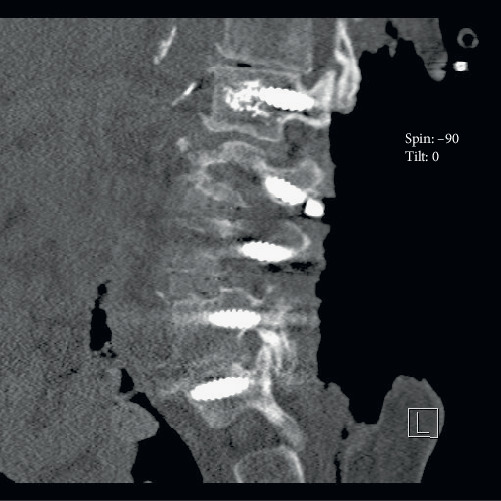
CT showing the placement of hardware prior to closing.

The patient elected for revisional surgery following a compression deformity at the L3 superior endplate with loss of previous L3 pedicle screw fixation. Bilateral L3 pedicle screws were replaced as well as replacement of previous L2 through S1 rods. At L2, the placement of new fenestrated pedicle screws was completed with the aid of fluoroscopic guidance. The methyl methacrylate delivery system was then used to introduce the vertebral body cement through the fenestrated pedicles. During the injection, a small amount of cement extravasated anteriorly to anterolaterally on the right, as visualized by fluoroscopy, and delivery was stopped. After CT confirmed acceptable placement of hardware, the screws were connected, rods were cross-linked, and closing procedures were followed (Figure 1).

Despite adequate healing of her surgical site and no other postoperative complications, the patient presented on postoperative day five with tachycardia (106 beats per minute), hypertension (189/49 mmHg), and rapidly desaturating oxygen levels (90% on room air). There were bilaterally diminished breath sounds on physical exam, and the patient was immediately given oxygen via nasal cannula. Arterial blood gas was notable for respiratory alkalosis (pH: 7.495, PaCO_2_: 32.8) and hypoxemia (PaO_2_: 55). Due to the patient's apparent hemodynamic instability and respiratory distress, she was transferred to the intensive care unit (ICU) with the presumptive diagnosis of postoperative pulmonary embolism.

**Figure 2:**
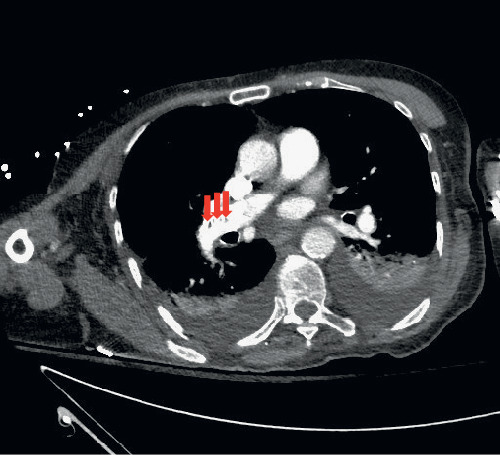
CT angiogram showing cement emboli (arrows) in the right lung.

Diagnostic CT angiogram (CTA) revealed intravasated cement in the right pulmonary artery, right upper lobar artery, right middle lobar artery, right middle segmental pulmonary artery branches, and multifocal areas within the upper subsegmental branches (Figure 2). The impression at this time was embolization of cement particles from the lumbar veins, which also showed CT evidence of intravasation. Additionally, mild bilateral pleural effusions and a right upper lobe consolidation were noted.

Due to the inorganic nature of the occluding material and mild hypoxemia, the consensus of the multidisciplinary team was to not initiate anticoagulation for the patient. In addition to using routine pulmonary toilet and incentive spirometry, the patient was given nebulized albuterol and ipratropium, 3 liters of oxygen via nasal cannula, and prophylactic cefepime and vancomycin. A positive airway pressure system and analgesics were ordered for use as needed.

Symptomatic management with careful observation of her vital signs and hemodynamic status was employed throughout her stay in the hospital. On postoperative day 8, the patient was discharged home on room air with opiates and nonopiate analgesics and instructions to follow-up outpatient in two weeks with neurosurgery.

## 3. Discussion

Cement pulmonary embolisms are frequent sequelae of operations involving PMMA, such as vertebroplasties, that often go undetected due to their asymptomatic nature [7]. Symptomatic CPEs are less common, and their management is controversial and generally based on physician preference [12]. However, most reported cases of symptomatic CPEs are treated with anticoagulation [9]. Our patient presented with dyspnea, hypoxemia, and tachycardia on postoperative day five, which raised a high clinical suspicion for acute biological pulmonary embolism [13]. However, routine CTA revealed widespread cement emboli. The decision of whether or not to use blood-thinning agents was difficult, and the risks and benefits of using blood-thinning agents were taken into consideration. Since cement is an inorganic substance, the only theoretical benefit of using agents that manipulate the clotting cascade is dissolution of thrombus deposited on the surface of cement particles and to prevent new clot from forming. It was decided that the risk of postoperative bleeding from anticoagulation or thrombolytic therapy was greater than the potential benefit. The physicians chose to treat with conservative, symptomatic management. With three days of supportive care and observation, the patient recovered and was discharged home without acute ramifications.

By treating conservatively, the risk of postoperative bleeding from anticoagulant or thrombolytic therapy was eliminated, and the patient avoided undergoing an invasive embolectomy. The case adds to the sparse literature on the management of symptomatic CPEs and helps illustrate that supportive care alone is an effective treatment modality [14]. We cannot draw any conclusions on the long-term prognosis of conservative therapy or risk of recurrence based on the acute resolution of her symptoms alone. Additional studies are needed to investigate any potential long-term complications of cement pulmonary emboli, especially in patients treated with anticoagulation versus symptomatic management, in order to develop guidelines on the standard of care for a CPE.

## Data Availability

No data were used to support this study.
